# Supplementary material for: Differences in Non-Pathogenic Lung-Colonizing Bacteria Among Patients with Different Types of Pneumonia: A Retrospective Study
Source: Microorganisms. 2025 Sep 9;13(9):2099. doi: 10.3390/microorganisms13092099 (PMC12472864; doi:10.3390/microorganisms13092099)
Supplement: Supplementary file 1 [file microorganisms-13-02099-s001.zip › Additional File.pdf]

Additional File

Table S1. Results of the univariable and multivariable logistic regression analyses of risk factors for sepsis in bacterial pneumonia patients.
Table S2. Results of the univariable and multivariable logistic regression analyses of risk factors for death in fungal pneumonia patients.
Table S3. Results of the univariable and multivariable logistic regression analyses of risk factors for the need for ventilator-assisted breathing in viral pneumonia patients.

Table S4. Baseline characteristics of all patients.

Table S5. The types and proportions of the common pathogenic bacteria in 321 confirmed bacterial pneumonia patients.
Table S6. The types and proportions of the common pathogenic fungi in 121 confirmed fungal pneumonia patients.

Table S7. Clinical characteristics of patients with and without bacterial pneumonia before and after PSM.
Table S8. Clinical characteristics of patients with and without fungal pneumonia before and after PSM.
Table S9. Clinical characteristics of patients with and without viral pneumonia before and after PSM.

Table S1. Results of the univariable and multivariable logistic regression analyses of risk factors for sepsis in bacterial pneumonia patients

**Characteristics**	**Univariable logistic regression**		**Multivariable logistic regression**	
	p	**OR**	***Adjust-p***	**OR**
**Age, years**	0.068	1.023(0.998-1.049)	0.016	1.073(1.013-1.136)
**Sex (Men)**	0.016	2.837(1.210-6.654)		
**Comorbidities**				
Hypertension	0.246	1.518(0.750-3.072)	0.026	6.631(1.251-35.154)
Diabetes	0.137	1.779(0.832-3.807)		
CHD	0.002	4.290(1.698-10.837)		
COPD	0.013	2.685(1.230-5.857)		
**Antibiotic use**				
Cumulative type of antibiotic use	<0.001	1.783(1.511-2.104)	<0.001	1.748(1.292-2.365)
Cumulative antibiotic use time	<0.001	1.122(1.079-1.167)		
**Disease severity**				
severe pneumonia	<0.001	28.664(12.656-64.918)	<0.001	28.513(6.201-131.113)

CHD: Coronary heart disease; COPD: Chronic obstructive pulmonary disease;

Table S2. Results of the univariable and multivariable logistic regression analyses of risk factors for death in fungal pneumonia patients

**Characteristics**	**Univariable logistic regression**		**Multivariable logistic regression**	
	p	**OR**	***Adjust-p***	**OR**
**Age, years**	0.183	1.033(0.985-1.082)		
**Sex (Men)**	0.324	2.219(0.456-10.810)		
**Comorbidities**				
Hypertension	0.045	3.644(1.030-12.900)		
Diabetes	0.166	2.543(0.679-9.526)		
CHD	<0.001	15.150(3.781-60.708)	0.034	7.865(1.170-52.882)
COPD	0.006	6.067(1.670-22.039)		
**Disease severity**				
severe pneumonia	0.001	39.545(4.803-325.583)		

CHD: Coronary heart disease; COPD: Chronic obstructive pulmonary disease;

Table S3. Results of the univariable and multivariable logistic regression analyses of risk factors for the need for ventilator-assisted breathing in viral pneumonia patients

**Characteristics**	**Univariable logistic regression**		**Multivariable logistic regression**	
	p	**OR**	***Adjust-p***	**OR**
**Age, years**	0.031	1.038(1.003-1.073)	0.030	5.750(1.185-27.905)
**Sex (Men)**	0.173	2.204(0.707-6.872)		
**Comorbidities**				
Hypertension	0.249	1.746(0.678-4.500)		
Diabetes	0.109	2.259(0.835-6.110)		
CHD	0.002	5.727(1.931-16.991)		
COPD	0.049	2.637(1.003-6.934)		
**Disease severity**				
severe pneumonia	<0.001	24.065(7.459-77.642)		

CHD: Coronary heart disease; COPD: Chronic obstructive pulmonary disease;

Table S4.Baseline characteristics of all patients.

Characteristics	All patients(n=483)
**Age, years**	63(50-71)
**Sex (Men)**	298(61.7%)
**Comorbidities**	
Hypertension	127(26.3%)
Diabetes	90(18.6%)
Coronary heart disease	36(7.5%)
Chronic obstructive pulmonary disease	63(13.0%)
**Laboratory Results**	
White blood cell count(10^9/L)	
<4	22(4.6%)
4-10	345(71.4%)
>10	116(24.0%)
Lymphocyte count(10^9/L)	1.46(0.99-2.00)
Monocyte count(10^9/L)	0.54(0.39-0.75)
Neutrophil count(10^9/L)	5.32(3.59-7.44)
Total bilirubin(μmol/L)	10.4(8.1-13.7)
Serum creatinine(μmol/L)	71(59-89)
Aspartate transaminase(U/L)	21(17-27)
Alanine aminotransferase(U/L)	16(11-26)
Direct bilirubin(μmol/L)	2.0(1.6-2.7)
Indirect bilirubin(μmol/L)	8.4(6.5-11.1)
Albumin(g/L)	35.2(31.3-38.7)
Creatine kinase-MB(ng/mL)	10(3-15)
High-sensitivity cardiac troponin I (ng/mL)	0.005(0.003-0.011)
Interleukin -2	0.38(0.01-0.99)
Interleukin -4	0.26 (0.01-0.63)
Interleukin -6	73.48(12.84-211.26)
Interleukin -10	8.78(2.46-38.18)
Tumor necrosis factor -α	1.15(0.01-6.08)
Interferon -γ	1.72 (0.01-7.31)
**Pneumonia**	
Bacterial pneumonia	321(66.5%)
Fungal pneumonia	121(25.1%)
Viral pneumonia	168(34.8%)
**Treatment**	
Cumulative type of antibiotics	2(1-4)
Cumulative number of days of antibiotic treatment	9(7-13)
Need oxygen	316(65.4%)
Assisted mechanical ventilation	69(14.3%)
**Prognosis**	
Severe pneumonia	73(15.1%)

Admitted to intensive care unit	52(10.8%)
Multiple organ dysfunction syndrome	50(10.4%)
Sepsis	31(6.4%)
Death	16(3.3%)

Cumulative type of antibiotics: cumulative type of antibiotics received by the patient during hospitalization; Cumulative number of days of antibiotic treatment: cumulative number of days of antibiotic treatment received by the patient during hospitalization

Table S5. The types and proportions of the common pathogenic bacteria in 321 confirmed bacterial pneumonia patients in the study.

**Pathogenic bacteria***	**Patients(n=321)**
Streptococcu pneumoniae	79(24.6%)
Pseudomonas aeruginosa	69(21.5%)
Haemophilus influenzae	58(18.1%)
Klebsiella pneumoniae	36(11.2%)
Acinetobacter baumannii	35(10.9%)
Staphylococcus aureus	29(9.0%)
Enterococcus faecium	27(8.4%)
Stenotrophomonas maltophilia	19(5.9%)
Tropheryma whipplei	19(5.9%)

* A small percentage of bacterial pneumonia patients were infected with two or more pathogenic bacteria simultaneously.

Table S6. The types and proportions of the common pathogenic fungi in 121 confirmed fungal pneumonia patients in the study.

**Pathogenic fungi***	**Patients(n=121)**
Candida	73(60.3%)
Pneumocystis jirovecii	27(22.3%)
Aspergillus	24(19.8%)
Cryptococcus	6(5.0%)
Talaromyces marneffei	3(2.5%)

* A small percentage of fungal pneumonia patients were infected with two or more pathogenic fungi simultaneously.

Table S7. Clinical characteristics of patients with and without bacterial pneumonia before and after PSM.

	Before PSM			After PSM		
**Characteristics**	**Patients with bacterial pneumonia (n=321)**	**Patients without bacterial pneumonia (n=162)**	***P*-value**	**Patients with bacterial pneumonia (n=154)**	**Patients without bacterial pneumonia (n=154)**	***Adjusted P*-value**
**Age, years**	64(53-72)	58(40-69)	<0.001	60(44-73)	58(43-69)	0.265
**Sex (Men)**	206(64.2%)	92(56.8%)	0.115	85(55.2%)	89(57.8%)	0.646
**Comorbidities**						
Hypertension	90(28.0%)	37(22.8%)	0.221	36(23.4%)	37(24.0%)	0.893
Diabetes	62(19.3%)	28(17.3%)	0.588	29(18.8%)	27(17.5%)	0.768
CHD	24(7.5%)	12(7.4%)	0.978	9(5.8%)	11(7.1%)	0.644
COPD	47(14.6%)	16(9.9%)	0.142	15(9.7%)	16(10.4%)	0.850
**Laboratory Results**						
WBC(10^9/L)	7.66(5.96-9.90)	7.38(5.90-9.87)	0.679	7.59(5.97-9.84)	7.42(6.00-9.86)	0.860
LY(10^9/L)	1.42(0.94-1.93)	1.56(1.15-2.11)	0.030	1.42(0.92-1.97)	1.55(1.15-2.11)	0.702
Mono(10^9/L)	0.54(0.40-0.75)	0.54(0.38-0.75)	0.679	0.54 (0.39-0.74)	0.54(0.38-0.75)	0.937
NE(10^9/L)	5.38(3.59-7.47)	5.28(3.49-7.35)	0.531	5.52(3.39-7.32)	5.28(3.53-7.35)	0.612
TD(μmol/L)	10.4(8.1-13.8)	10.4(7.9-13.6)	0.641	10.2(8.1-12.9)	10.4(7.9-13.7)	0.576
Cr(μmol/L)	71(60-89)	71(58-87)	0.419	68(58-84)	72(59-87)	0.328
AST(U/L)	21(17-26)	21(17-30)	0.708	21(17-27)	21(17-30)	0.717
ALT(U/L)	15(11-26)	17(11-28)	0.266	15(10-24)	16(11-29)	0.090
DD(μmol/L)	2.1(1.6-2.8)	2.0(1.5-2.7)	0.318	2.0(1.6-2.7)	2.0(1.5-2.7)	0.522
ID(μmol/L)	8.2(6.6-11.0)	8.5(6.3-11.3)	0.956	8.0(6.4-10.2)	8.5(6.3-11.3)	0.337
ALB(g/L)			0.300			0.880
<30	64(19.9%)	26(16.0%)		27(17.5%)	26(16.9%)	
≥30	257(80.1%)	136(84.0%)		127(82.5%)	128(83.1%)	

CK-MB(ng/mL)	10(3-15)	10(3-15)	0.997	10(3-15)	10(3-15)	0.966
cTnI-HS(ng/mL)	0.005(0.003-0.012)	0.004(0.002-0.009)	0.055	0.005(0.003-0.012)	0.005(0.002-0.009)	0.177
IL-2(pg/mL)	0.46(0.01-1.00)	0.27(0.01-0.88)	0.092	0.50(0.01-1.02)	0.29(0.01-0.88)	0.055
IL-4(pg/mL)	0.28(0.01-0.64)	0.25(0.01-0.62)	0.952	0.33(0.01-0.72)	0.25(0.01-0.64)	0.620
IL-6(pg/mL)	86.95(15.15-229.26)	58.46(6.91-184.26)	0.022	88.76(14.95-262.59)	61.93(6.74-188.61)	0.034
IL-10(pg/mL)	9.32(2.69-34.65)	8.37(2.10-38.84)	0.847	10.37(3.19-39.23)	8.37(2.10-38.33)	0.530
TNF-α(pg/mL)	1.43(0.01-6.12)	0.89(0.01-6.18)	0.619	1.99(0.01-6.63)	0.70(0.01-6.05)	0.291
IFN-γ(pg/mL)	1.55(0.01-7.08)	2.61(0.01-7.91)	0.671	2.26(0.01-7.57)	2.42(0.01-7.93)	0.922

PSM: propensity score matching; CHD: Coronary heart disease; COPD: Chronic obstructive pulmonary disease; PCT: Procalcitonin; WBC: White blood cell count; LY: Lymphocyte count; Mono: Monocyte count; NE : Neutrophil count; TD: Total bilirubin; Cr: Serum creatinine; AST: Aspartate transaminase; ALT: Alanine aminotransferase; DD: Direct bilirubin; ID: Indirect bilirubin; ALB: Albumin; CK-MB: Creatine kinase-MB; cTnI-HS：High-sensitivity cardiac troponin I; IL: Interleukin; TNF: Tumor necrosis factor; IFN: Interferon.

Table S8. Clinical characteristics of patients with and without fungal pneumonia before and after PSM.

	Before PSM			After PSM		
**Characteristics**	**Patients with fungal pneumonia (n=121)**	**Patients without fungal pneumonia (n=362)**	***P*-value**	**Patients with fungal pneumonia (n=118)**	**Patients without fungal pneumonia (n=118)**	***Adjusted P*-value**
**Age, years**	65(58-74)	62(48-69)	0.001	65(57-73)	66(55-72)	0.682
**Sex (Men)**	83(68.6%)	215(59.4%)	0.071	80(67.8%)	79(66.9%)	0.890
**Comorbidities**						
Hypertension	33(27.3%)	94(26.0%)	0.778	32(27.1%)	31(26.3%)	0.883
Diabetes	24(19.8%)	66(18.2%)	0.695	23(19.5%)	19(16.1%)	0.496
CHD	14(11.6%)	22(6.1%)	0.046	11(9.3%)	9(7.6%)	0.640
COPD	24(19.8%)	39(10.8%)	0.010	22(18.6%)	20(16.9%)	0.734
**Laboratory Results**						
WBC(10^9/L)	7.87(6.19-10.33)	7.43(5.84-9.70)	0.113	7.78(6.13-10.31)	7.41(5.84-8.90)	0.106
LY(10^9/L)	1.09(0.62-1.66)	1.56(1.18-2.03)	0.000	1.12(0.62-1.71)	1.56(1.18-1.98)	<0.001
Mono(10^9/L)	0.58(0.42-0.83)	0.53(0.39-0.74)	0.295	0.57(0.41-0.82)	0.53(0.40-0.71)	0.475
NE(10^9/L)	6.07(4.03-8.54)	5.05(3.38-7.15)	0.002	6.07(3.98-8.45)	5.03(3.38-6.61)	0.005
TD(μmol/L)	9.8(7.7-14.1)	10.5(8.3-13.7)	0.252	9.9(7.7-14.1)	10.5(8.8-13.7)	0.251
Cr(μmol/L)	78(62-94)	69(58-84)	0.003	78(62-94)	69(57-84)	0.014
AST(U/L)			0.021			0.034
>40	22(18.2%)	37(10.2%)		21(17.8%)	10(8.5%)	
≤40	99(81.8%)	325(89.8%)		97(82.2%)	108(91.5%)	
ALT(U/L)			0.048			0.138
>40	23(19.0%)	43(11.9%)		21(17.8%)	13(11.0%)	
≤40	98(81.0%)	319(88.1%)		97(82.2%)	105(89.0%)	
DD(μmol/L)	2.0(1.5-3.1)	2.0(1.6-2.7)	0.394	2.0(1.5-3.1)	2.2(1.7-2.7)	0.802
ID(μmol/L)	7.6(5.9-11.1)	8.5(6.7-11.2)	0.095	7.7(5.8-11.0)	8.5(6.9-11.3)	0.166

ALB(g/L)			<0.001			0.001
<30	39(32.2%)	51(14.1%)		38(32.2%)	16(13.6%)	
≥30	82(67.8%)	311(85.9%)		80(67.8%)	102(86.4%)	
CK-MB(ng/mL)	9(3-18)	10(3-15)	0.522	10(3-19)	11(4-14)	0.914
cTnI-HS(ng/mL)			0.034			0.023
>ULN	29(24.0%)	56(15.5%)		26(22.0%)	13(11.0%)	
≤ULN	92(76.0%)	306(84.5%)		92(78.0%)	105(89.0%)	
IL-2(pg/mL)	0.26(0.01-0.77)	0.46(0.01-1.01)	0.047	0.27(0.01-0.76)	0.42(0.01-1.04)	0.058
IL-4(pg/mL)	0.18(0.01-0.62)	0.31(0.01-0.64)	0.074	0.18(0.01-0.61)	0.36(0.01-0.65)	0.050
IL-6(pg/mL)	67.64(10.04-184.84)	78.50(13.86-218.74)	0.365	69.08(9.80-190.29)	85.75(15.98-230.39)	0.283
IL-10(pg/mL)	6.37(2.12-21.84)	10.31(2.74-40.64)	0.023	6.37(2.11-22.30)	15.69(3.48-41.47)	0.009
TNF-α(pg/mL)	0.03(0.01-3.85)	2.01(0.01-7.06)	0.001	0.23(0.01-4.08)	2.15(0.01-7.82)	0.004
IFN-γ(pg/mL)	0.36(0.01-5.05)	2.33(0.01-7.87)	0.002	0.38(0.01-5.23)	2.04(0.01-7.91)	0.008

PSM: propensity score matching; CHD: Coronary heart disease; COPD: Chronic obstructive pulmonary disease; PCT: Procalcitonin; WBC: White blood cell count; LY: Lymphocyte count; Mono: Monocyte count; NE : Neutrophil count; TD: Total bilirubin; Cr: Serum creatinine; AST: Aspartate transaminase; ALT: Alanine aminotransferase; DD: Direct bilirubin; ID: Indirect bilirubin; ALB: Albumin; CK-MB: Creatine kinase-MB; cTnI-HS：High-sensitivity cardiac troponin I; ULN: upper limit of normal value ;IL: Interleukin; TNF: Tumor necrosis factor; IFN: Interferon.

The upper limit of normal value of cTnI-HS in this study is 0.0175 ng/mL.

Table S9. Clinical characteristics of patients with and without viral pneumonia before and after PSM.

	Before PSM			After PSM		
**Characteristics**	**Patients with viral pneumonia (n=168)**	**Patients without viral pneumonia (n=315)**	**P-value**	**Patients with viral pneumonia (n=160)**	**Patients without viral pneumonia (n=160)**	***Adjusted P*-value**
**Age, years**	65(53-72)	62(48-69)	0.022	65(51-72)	63(51-71)	0.542
**Sex (Men)**	116(69.0%)	182(57.8%)	0.015	108(67.5%)	102(63.8%)	0.480
**Comorbidities**						
Hypertension	44(26.2%)	83(26.3%)	0.970	39(24.4%)	46(28.8%)	0.376
Diabetes	32(19.0%)	58(18.4%)	0.864	29(18.1%)	28(17.5%)	0.884
CHD	18(10.7%)	18(5.7%)	0.046	13(8.1%)	15(9.4%)	0.692
COPD	34(20.2%)	29(9.2%)	0.001	26(16.3%)	25(15.6%)	0.879
**Laboratory Results**						
WBC(10^9/L)	8.17(6.15-10.72)	7.38(5.84-9.67)	0.086	8.15(6.15-10.72)	7.51(5.78-9.86)	0.378
LY(10^9/L)	1.35(0.86-1.77)	1.55(1.08-2.04)	0.002	1.35(0.84-1.79)	1.57(1.08-2.11)	0.003
Mono(10^9/L)	0.57(0.42-0.81)	0.53(0.38-0.74)	0.077	0.56(0.42-0.81)	0.55(0.42-0.77)	0.659
NE(10^9/L)	5.92(3.72-8.02)	5.03(3.48-7.14)	0.030	5.92(3.66-7.86)	5.09(3.41-7.28)	0.140
TD(μmol/L)	10.2(7.9-14.2)	10.5(8.2-13.3)	0.740	10.2(7.9-14.1)	10.4(8.1-14.4)	0.668
Cr(μmol/L)	73(61-90)	70(58-87)	0.126	73(62-90)	72(58-90)	0.444
AST(U/L)	22(18-30)	21(17-26)	0.288	22(18-29.75)	23(18-27)	0.779
ALT(U/L)	18(11-28)	15(11-25)	0.100	18(11-27)	15(11-28)	0.448
DD(μmol/L)	2.1(1.5-3.0)	2.0(1.6-2.7)	0.779	2.0(1.5-3.0)	2.0(1.6-2.8)	0.915
ID(μmol/L)	8.0(6.0-11.3)	8.5(6.7-11.1)	0.268	8.0(6.0-11.1)	8.4(6.6-11.5)	0.253
ALB(g/L)			0.002			0.013
<30	44(26.2%)	46(14.6%)		42(26.3%)	24(15.0%)	
≥30	124(73.8%)	269(85.4%)		118(73.8%)	136(85.0%)	
CK-MB(ng/mL)	10(3-16)	10(3-15)	0.669	10(3-16)	10(3-15)	0.611

cTnI-HS(ng/mL)			0.389			0.765
>ULN	33(19.6%)	52(16.5%)		28(17.5%)	26(16.3%)	
≤ULN	135(80.4%)	263(83.5%)		132(82.5%)	134(83.8%)	
IL-2(pg/mL)	0.27(0.01-0.86)	0.47(0.01-1.00)	0.147	0.31(0.01-0.93)	0.48(0.01-1.00)	0.338
IL-4(pg/mL)	0.14(0.01-0.57)	0.31(0.01-0.66)	0.018	0.14(0.01-0.54)	0.21(0.01-0.64)	0.341
IL-6(pg/mL)	70.98(14.88-178.31)	78.16(10.99-227.52)	0.367	70.98(14.68-181.87)	83.80(7.87-226.83)	0.671
IL-10(pg/mL)	7.78(2.57-29.99)	10.22(2.41-40.73)	0.292	7.78(2.41-29.99)	7.14(2.04-37.73)	0.701
TNF-α(pg/mL)	0.36(0.01-5.35)	1.92(0.01-7.07)	0.006	0.42(0.01-5.45)	1.36(0.01-6.88)	0.087
IFN-γ(pg/mL)	0.62(0.01-4.88)	2.76(0.01-8.12)	0.002	0.62(0.01-5.02)	2.37(0.01-8.90)	0.019

PSM: propensity score matching; CHD: Coronary heart disease; COPD: Chronic obstructive pulmonary disease; PCT: Procalcitonin; WBC: White blood cell count; LY: Lymphocyte count; Mono: Monocyte count; NE : Neutrophil count; TD: Total bilirubin; Cr: Serum creatinine; AST: Aspartate transaminase; ALT: Alanine aminotransferase; DD: Direct bilirubin; ID: Indirect bilirubin; ALB: Albumin; CK-MB: Creatine kinase-MB; cTnI-HS：High-sensitivity cardiac troponin I; ULN: upper limit of normal value; IL: Interleukin; TNF: Tumor necrosis factor; IFN: Interferon.

The upper limit of normal value of cTnI-HS in this study is 0.0175 ng/mL
