# Supplementary figures and images for: Differences in Non-Pathogenic Lung-Colonizing Bacteria Among Patients with Different Types of Pneumonia: A Retrospective Study
Source: Microorganisms. 2025 Sep 9;13(9):2099. doi: 10.3390/microorganisms13092099 (PMC12472864; doi:10.3390/microorganisms13092099)

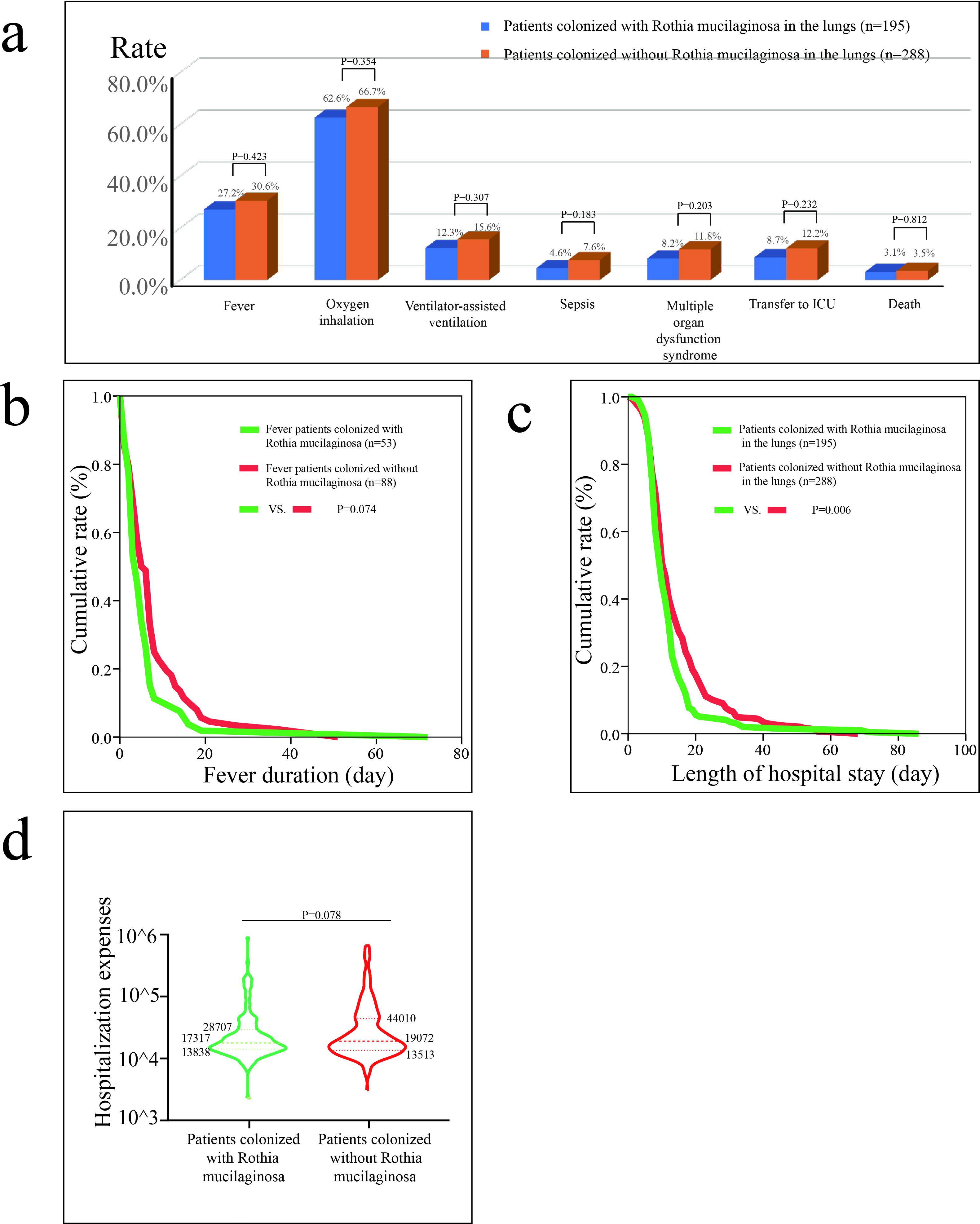

Supplement: Supplementary file 1 [file microorganisms-13-02099-s001.zip › Figure S5.tif]

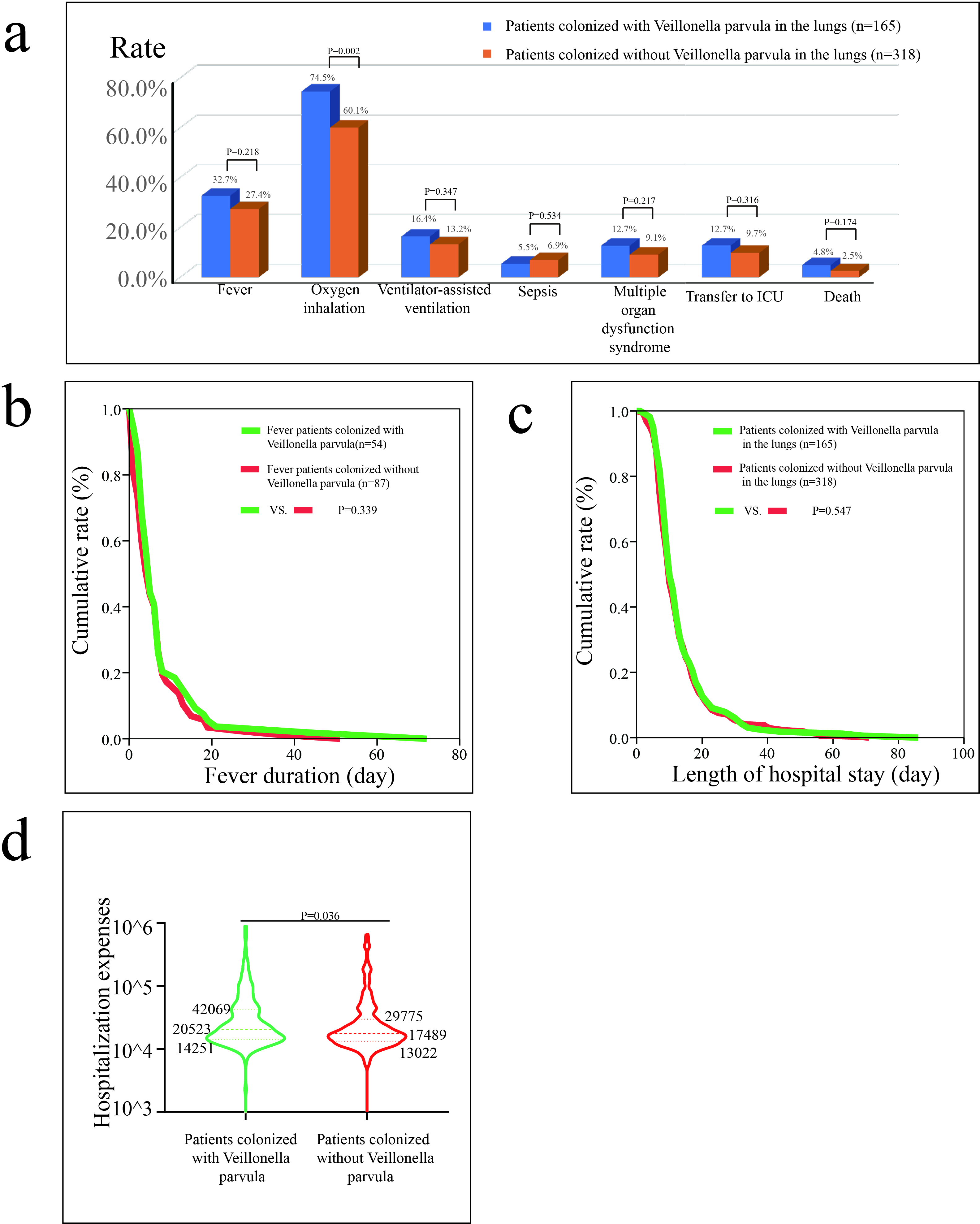

Supplement: Supplementary file 1 [file microorganisms-13-02099-s001.zip › Figure S6.tif]

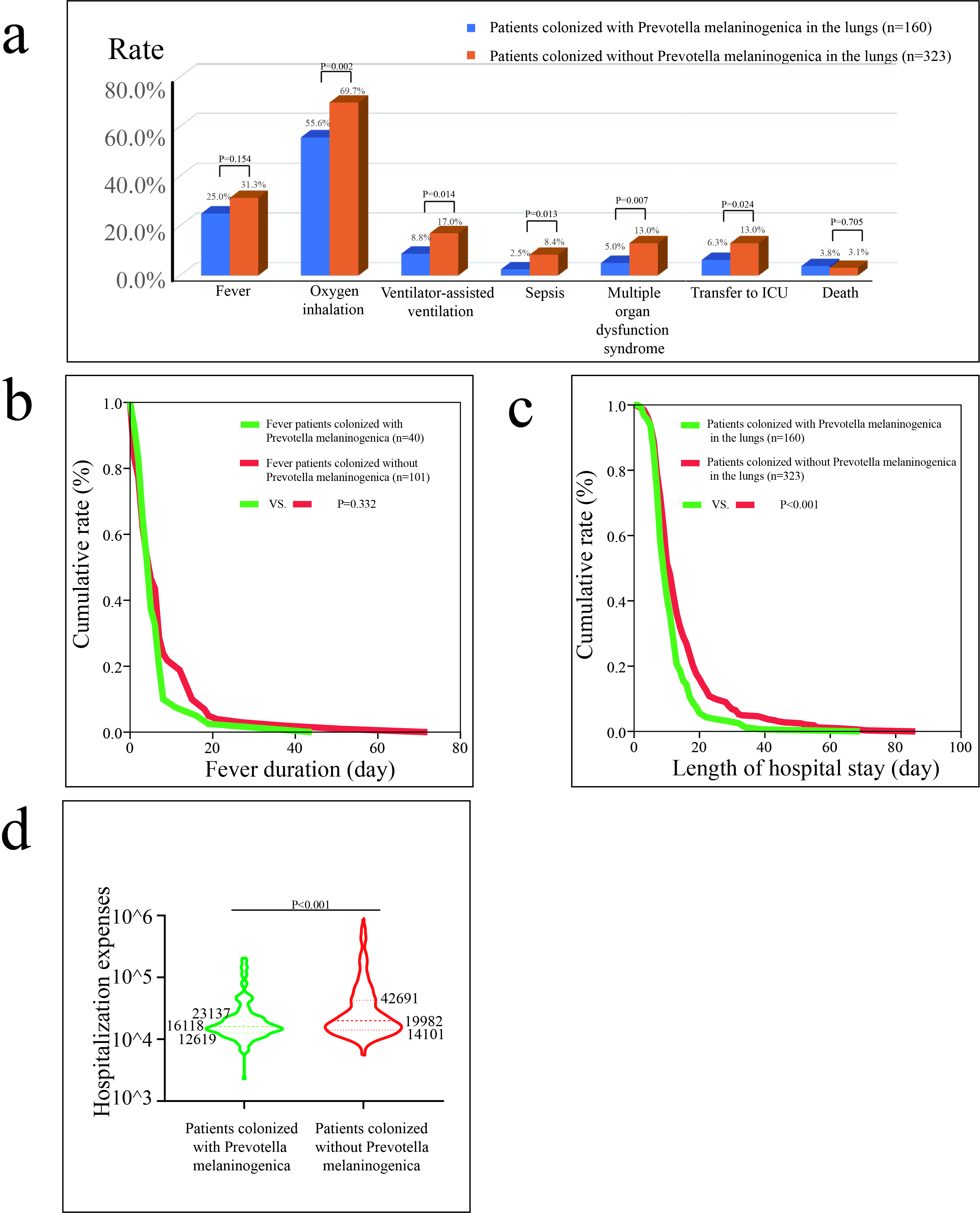

Supplement: Supplementary file 1 [file microorganisms-13-02099-s001.zip › Figure S7.tif]
